# Changes in Resilience in Students of Occupational Therapy After Their First Exposure to Practice Placement Education

**DOI:** 10.3389/fpsyg.2021.658187

**Published:** 2021-05-07

**Authors:** María Del Carmen Rodríguez-Martínez, Abel Toledano-González, José-Matías Triviño-Juárez, Begoña Polonio-López, Antonio Segura-Fragoso, Olga López-Martín, Pablo Cantero-Garlito, Marta Rodríguez-Hernández, Ana-Isabel Corregidor-Sánchez, Dulce Romero-Ayuso

**Affiliations:** ^1^Department of Physical Therapy, Faculty of Health Sciences, University of Malaga, Málaga, Spain; ^2^Department of Psychology, Faculty of Health Sciences, University of Castilla La Mancha, Talavera De La Reina, Spain; ^3^Neurological Disabilities Research Institute, Albacete, Spain; ^4^Primary Care Center Zaidín South, Andalusian Health Service, Granada, Spain; ^5^Department of Nursing, Physical Therapy and Occupational Therapy, Faculty of Health Sciences, University of Castilla La Mancha, Talavera de la Reina, Spain; ^6^Department of Medical Sciences, Faculty of Health Sciences, University of Castilla La Mancha, Talavera de la Reina, Spain; ^7^Department of Physical Therapy, Faculty of Health Sciences, University of Granada, Granada, Spain

**Keywords:** resilience (psychological), college student, clinical practice, occupational therapy, personal development

## Abstract

**Introduction:** Resilience is a multidimensional and dynamic construct associated with positive growth and the capacity to transform stressful and negative factors into opportunities of personal development and self-improvement when faced with difficult experiences. The resilience process of each individual integrates multiple analysis levels, which range from genetic-environmental interactions to a complex process of adaptation between the individual and his/her family, friends, co-workers, society, and culture.

**Objective:** To determine whether resilience improves in students of occupational therapy when exposed for the first time to practice placement education.

**Methodology:** Quasi-experimental, prospective, observational, multi-center study with a sample composed of students from the Degree of Occupational Therapy of the public universities of Málaga (UMA) and Castilla-La Mancha (UCLM) (Spain). Two weeks prior to the beginning of the practice education period, the participants completed a questionnaire that included sociodemographic data and the area of their internships. They were also given the Spanish version of the Connor-Davidson's resilience scale (CD-RISC). All these instruments were also completed 1 week after the end of the clinical practice.

**Results:** There were statistically significant differences between the variables that make up resilience and the different internship areas. On the other hand, there was a significant improvement of global resilience after the clinical practice period, in both women (13.85 points; *p* < 0.001) and men (7.72 points; *p* < 0.035), when the internship area was not considered.

**Conclusions:** The results show that resilient students are more optimistic and work to improve a situation beyond doing simply what is expected of them, knowing how to control their feelings. This is beneficial for students in practice education, since, during these, they face difficult situations that require a resilient pattern, which helps reduce stress and the burnout syndrome.

## Introduction

Emotional skills such as mood, feelings, and emotions, are an important factor in the adequate professional performance of both the individual and other people's professionals. These aspects are closely related to the learning process of students during their practice placement education (Bennis et al., 2001; Garner, 2010; Arghode, 2013). In the psychological field, resilience can be understood as a personality quality observed in the adaptive response to adverse or risky circumstances (Piña López, 2015). Resilience is understood as a dynamic and multidimensional construct that refers to the capacity of an individual to overcome difficult situations and face stress as a result of adaptation (Connor and Davidson, 2003; Hecht et al., 2014). Aspects of building resilience such as resilience are connected to positive growth and overcoming challenges (Luthar and Brown, 2007; Masten, 2007), transforming stressors and negatives into opportunities for personal development and overcoming difficult experiences (Hecht et al., 2014). It includes two relevant aspects: (1) significant exposure to risk, and (2) evidence of positive adaptation despite serious threats to development (Lutha and Cicchetti, 2000; Bonanno, 2004; Masten, 2007; González-Torres and Artuch, 2014). Resilience begins to develop before the university period, through the changes and challenges of the previous stages that allow the development of emotional regulation strategies lead to the development of resilience (Mestre et al., 2017). Therefore, it is a way of facing adversity that promotes the use of cognitive and behavioral strategies throughout life (Sinclair and Wallston, 2004).

There are six factors that explain the structure of resilience: self-perception, perception of the future, structured style, social competence, family cohesion, and social resources (Friborg et al., 2005). Thus, self-perception is defined as the state of a person who is self-aware; perception of the future is an individual's perspective on the future; structured style refers to personal attributes, such as self-confidence, strength, and self-discipline; social competence is defined as the support received toward an individual; family cohesion is the harmony between an individual and his or her relatives; and social resources measure the degree and quality of social support received by an individual (Friborg et al., 2005).

Other authors also point out different protection factors (internal and external) that allow managing in a positive way any situation regardless of adversity (González-Torres and Artuch, 2014). The internal protection factors include high level of intelligence, adequate development of confrontation strategies, optimism, problem solving, and self-regulation (González-Torres and Artuch, 2014), as well as parenting styles, family structure, and the cohesion and relationships between teachers and parents (García-Vesga and Domínguez-de la Ossa, 2013). The external protection factors include: the care for others and the affection received, expectations of the environment, participation, and contribution in the community (González-Torres and Artuch, 2014).

Therefore, the resilience process of each person integrates multiple analysis levels, which range from genetic-environmental interactions to a complex process of adaptation between the individual and her/his family, friends, co-workers, society, and culture (O'Dougherty and Masten, 2013). Thus, resilience as a protection mechanism allows some people to face real risks above the conventional expectations, which explains the individual differences in adaptation processes (Rutter, 2006; Masten, 2007). A pattern of resilience has been identified, in which positive adaptive responses to tragedy, trauma and adversity are activated, allowing for the overcoming of stressors throughout life, such as the loss of a loved one or someone close to them (Newman, 2005). Therefore, resilience would function as a self-regulating mechanism that protects the personal systems from negative consequences in difficult stages of life (Masten, 2007).

From the labor or professional point of view, another aspect that may influence resilience is professional identity, which is established when students learn theoretical knowledge within the curriculum, and increases during the clinical internships, where they learn to connect the theoretical and practical knowledge while they attend to the clients (Ikiugu and Smallfield, 2015). The capacity to apply the theoretical knowledge in the practice placement education, including the reference frameworks and the duty-centred models, is fundamental for the development of professional identity and resilience (Hanson, 2015). In a study carried out with nursing students, it was observed that resilience involved the capacity to be oriented toward the future (Glass, 2007). Such work found that the relationship between hopeful and optimistic thoughts and feelings in professional actions influence the improvement of the levels of resilience and professional satisfaction.

Likewise, hope was perceived as an essential component of resilience, considering personal and professional resilience as an integral part of well-being (Glass, 2007). A different study, conducted with faculty members, showed that those with a strong professional pattern (professional environment) also had high self-esteem, confidence, resilience, and were flexible and goal-oriented, allowing them to be more optimistic and able to make the necessary changes to adapt to the context and apply new learning strategies (Abu-Tineh, 2011). These strategies can also be transferred to the personal sphere.

Although there are different studies in the field of healthcare that tackle the topic of resilience (Glass, 2007; O'Dougherty and Masten, 2013; Stoffel, 2014), there is not enough scientific evidence in the field of occupational therapy. Our initial hypothesis was that the resilience is improved by the perform of clinical practices, since these are the most similar to a professional experience. Stoffel pointed out the need to analyze resilience in occupational therapy professionals (Stoffel, 2014). This is in line with what other authors have stated about the importance of exploring in the curriculum. Based on the strategies that students adopt for correct performance in professional practice, along with the strategies that allow them to develop their resilience in the professional context (Scanlan et al., 2017). In accordance with the above mentioned, the aim of the present study was to determine whether there is an improvement in the resilience of students of occupational therapy after their first exposure to practice placement education. This starting point is important for designing methodological strategies that stimulate resilience so that students can have the skills to cope with difficult situations when they finish college and enter the labor market.

## Methodology

### Design

The design of the present study is a multi-center, quasi-experimental, pre-post study during their first exposure to clinical practice.

### Participants

Participants were students of the Occupational Therapy Degree from the public universities of Málaga (UMA) and Castilla-La Mancha (UCLM), both located in Spain. The students who participated were a total of 174 with an average age of 21.34, mostly women due to the high percentage of people enrolled in health-related degrees 84.5% (Table 1). All of them were recruited during the 2016–2017 academic year and were selected through a non-probabilistic selection process after verifying that they met the inclusion and exclusion criteria assigned during the research (not having received any previous practical training in addition to signing the informed consent form). Their participation was entirely voluntary. Moreover, the vast majority of participants were Spanish (96.6%). With respect to the UMA, in the academic year 2016–2017 there was only a curriculum in force, in which the students began their practice placement education in the 3rd year. With regard to the UCLM, in the academic year 2016–2017 there were two different curricula: a new curriculum, in which the students began their clinical internships in the 2nd year (OT Degree Plan 382), and an old, almost extinct curriculum, in which they began their practice education in the 3rd year (OT Degree Plan 311). Both in the UMA and in the UCLM, the first practical stay took place in the second semester of the academic year, and its duration ranged between 4 and 6 weeks. The practice placement education that takes place in the participating Spanish universities are regulated in terms of content and current syllabuses, with the aim of ensuring that students carry out their placements with the same learning guarantees.

**Table 1:** Demographic characteristics of subjects (*N* = 174).

		***n***	**%**
**Nationality**			
	Spanish	168	96.60
	Others	6	3.40
**Sex**			
	Female	147	84.50
	Male	27	15.50
**University**			
	UCLM	105	60.30
	UMA	69	39.70
**Course**			
	Second	54	31
	Third	120	69

*UCLM, University of Castilla-La Mancha; UMA, University of Málaga*.

### Procedure and Instruments

The participants completed a questionnaire 2 weeks before beginning the practical period, which included sociodemographic data (age, nationality, gender, university, and course) and the area in which they were going to carry out their internships: attention to children, physical rehabilitation, mental health, geriatrics and gerontology, or other non-conventional areas (social exclusion, gender violence, prisons). Along with this questionnaire, the Connor-Davidson's resilience scale (CD-RISC) (Connor and Davidson, 2003) was applied in its Spanish version, provided by these authors, which was completed again by the participants 1 week after the end of their practical internships. The scale used consists of 25 items. Each item is scored according to a Likert scale of 5 answers, from 0 (totally disagree) to 4 (totally agree), where the individual evaluates how she/he felt during the last month. Based on the answers provided, the total score ranges between 0 and 100 points; thus, the higher the score, the higher the level of resilience. The Cronbach'α for the global scale was 0.89 (general population), showing an adequate internal consistency (Connor and Davidson, 2003). In studies carried out with Spanish populations, the instrument has shown an adequate internal consistency, with a Cronbach's α of 0.86 (García-León et al., 2019). Although some authors support the hypothesis of the unidimensionality of CD-RISC (García-León et al., 2019), others have found, in Spanish populations, through a principal component analysis, three factors or dimensions of resilience (Manzano-García and Ayala Calvo, 2013): “tough personality” characterized by the dynamic organization in the individual of those systems that determine his or her characteristic behavior and thinking (items 4, 12, 14, 15, 16, 17, 18, 23, and 24), “resources” tools that are latent and unknown to the individual until now (items 1, 2, 5, 11, 13, 22, and 25), and “optimism” characterized by the ability to face difficulties with good cheer, perseverance, and hope (items 6, 7, 8, 10, 19, 20, and 21). After such analysis, items 3 and 9 were excluded (Manzano-García and Ayala Calvo, 2013). In view of this situation and following the criteria of Manzano-García (Manzano-García and Ayala Calvo, 2013), in the study it was decided to take into account the overall score of CD-RISC, as well as the score of each of these dimensions.

### Statistical Analysis

Descriptive statistics were used for the quantitative and qualitative variables at the beginning shown in Table 1. Likewise, we analyzed the differences in the sociodemographic variables between the students who completed both pre- and post- measures those who completed the pre- measures, but not the post- measure, using the *t*-test for independent samples and the Pearson chi-square test or the Fisher exact test when the expected values in any of the cells of the contingency tables were <5 (Martínez-González et al., 2009). To determine changes in participants' resistance after exposure to the practical training, we analyzed differences in the overall score and in each of the three factors or dimensions of the CD-RISC (resistant personality, resources, and optimism), using the *t*-test for paired samples, without first considering the area of the practice education training, and then separating on the basis of this variable. The statistical analysis was performed using IBM SPSS v24. The level of statistical significance was established for a value of *p* < 0.05.

### Ethical Aspects

All the participants gave their informed consent, and the objectives of the study were explained to them. The gathered data were treated in compliance with the declaration of Helsinki and the Organic Law of Regulation of the Automated Treatment of Personal Data, for research purposes, guaranteeing their protection and confidentiality. The study was approved by the Ethics Committee of Scientific Research of the Integrated Area of Healthcare in Talavera de la Reina (CEIC code: 6/2017) and by the Ethics Committee of Experimentation of the University of Malaga (CEUMA: 86-2016-H). Likewise, the authors of the original CD-RISC questionnaire authorized the use of this instrument in the present study.

## Results

The number of participants who completed the CD-RISC after finishing their practice placement education stay was 156 (89.65% of the initial sample). There were no significant differences in the sociodemographic variables (age, nationality, gender, university, and course) between the students who completed the CD-RISC before and after their internships (*n* = 156) and those who only did so before their internships (*n* = 18) (Tables 1, 2).

**Table 2:** Differences between the groups of students who completed and not the follow-up.

		**Complete the follow up**		**No follow-up**		
		**mean**	**(SD)**	**mean**	**SD**	***p*-value**
Age		21.36	(2.63)	21.17	(1.62)	0.666^*^
		*n*	%	*n*	%	
**Nationality**						
	Spanish	151	(96.80)	17	(94.40)	0.486^**^
	Others	5	(3.20)	1	(5.60)	
**Sex**						
	Female	131	(84)	16	(88.90)	0.743^**^
	Male	25	(16)	2	(11.10)	
**University**						
	UCLM	91	(58.30)	14	(77.80)	0.132^**^
	UMA	65	(41.70)	4	(22.20)	
**Course**						
	Second	46	(29.50)	8	(44.40)	0.303^***^
	Third	110	(70.50)	10	(55.60)	

* *p-value unpaired t-test*;

** *p-value Fisher exact test*;

*** *p-value chi-square test: UCLM, University of Castilla-La Mancha; UMA. University of Malaga*.

With respect to the practice education area, 5.10% of the students (*n* = 8) carried out their stay in attention to children, 40.40% (*n* = 63) in geriatrics and gerontology, 19.90% (*n* = 31) in mental health, 29.50% (*n* = 46) in physical rehabilitation, and 5.10% (*n* = 8) in other non-conventional areas (social exclusion, gender violence, prisons).

### Changes in Global Resilience After the First Period of Practice Education Training

With respect to the female students, disregarding the area of practice education training, the results show a significant improvement of global resilience after the internship period (13.85 points; *p* < 0.001) (Table 3). Taking the internship area into account, an improvement was found in the score of global resilience in those female students who spent their practical stay physical rehabilitation we found a mean score before (57.80) and a mean score after clinical practice of (74.05), with a mean difference of (16.25 points; *p* < 0.001), mental health we found a mean score before (59.71) and a mean score after clinical practice of (74.86), with a mean difference of (15.15 points; *p* < 0.001), in the field of geriatrics and gerontology we start from an average score of (61.96), reaching a score after the clinical practice of (74.41), so the difference in averages is (12.45 points; *p* < 0.001), and finally, in attention to children starting from an average score of (67) and achieving a score after clinical practice of (75.50), the difference between average scores is (8.50 points; *p* = 0.028) (Table 3). There were no significant changes in the participants who carried out their internships in other non-conventional areas.

**Table 3:** Practical training PRE-POST scores of global resilience (CD-RISC 25) in female students (*n* = 131).

	**CD-RISC Global PRE** **Mean (SD)**	**CD-RISC Global POST** **Mean (SD)**	**Difference between** **means (SD)**	***p*-value** **(paired *t*-test)**	**95% CI**
**General** (*n =* 131)	60.52 (14.93)	74.37 (9.81)	13.85 (12.80)	** <0.001**	11.63–16.06
**Practice Education Areas**					
Children (*n =* 6)	67 (10.20)	75.50 (10.17)	8.50 (6.77)	**0.028**	1.39–15.61
Physical rehabilitation (*n =* 41)	57.80 (13.81)	74.05 (11.42)	16.25 (10.98)	** <0.001**	12.78–19.71
Mental health (*n =* 28)	59.71 (14.10)	74.86 (8.40)	15.15 (12.80)	** <0.001**	10.18–20.11
Geriatrics and gerontology (*n =* 49)	61.96 (16.52)	74.41 (9.67)	12.45 (13.80)	** <0.001**	8.49–16.41
Other areas (*n =* 7)	64 (16.41)	73 (7.77)	9 (18.20)	0.239	−7.84–25.84

*SD, standard deviation; 95% CI, 95% Confidence Interval*.

*The values marked in bold are the result of the analyses shown in the article*.

*These references are significant at a significance of p < 0.05*.

With respect to the male students, disregarding the area of practical training, the results show a significant improvement of global resilience after the practice placement education period (7.72 points; *p* < 0.035) (Table 4). Taking the internship area into account, an improvement was found in the score of global resilience in those male students who spent their practical stay in geriatrics and gerontology a pre-practice score of (62.36), a post-practice score of (72.57) and a mean score difference of (10.21 points; *p* = 0.032) are collected. There were no significant changes in any of the other areas (Table 4).

**Table 4:** Practical training PRE-POST scores of global resilience (CD-RISC 25) in male students (*n* = 25).

	**CD-RISC Global PRE** **Mean (SD)**	**CD-RISC Global POST** **Mean (SD)**	**Difference between** **means (SD)**	***p*-value** **(paired *t*-test)**	**CI 95%**
**General** (*n =* 25)	64.56 (18.50)	72.28 (9.23)	7.72 (17.27)	**0.035**	0.60–14.85
**Practice Education Areas**					
Children (*n =* 2)	68 (32.53)	55 (7.07)	−13 (25.46)	0.602	−241.71–215.71
Physical rehabilitation (*n =* 5)	72.60 (22.37)	74.20 (7.56)	1.60 (17.64)	0.849	−20.31–23.51
Mental health (*n =* 3)	65 (14.53)	78 (8)	13 (12.12)	0.204	−17.12–43.12
Geriatrics and gerontology (*n =* 14)	62.36 (17.56)	72.57 (8.36)	10.21 (15.94)	**0.032**	1.01–19.42
Other areas (*n =* 1)^*^	NA	NA	NA	NA	NA

* *The correlation and T cannot be calculated because the sum of the weights of the cases is ≤1; NA, Not applicable. The values marked in bold are the result of the analyses shown in the article*.

*These references are significant at a significance of p < 0.05*.

### Changes in the Dimensions of Resilience (Tough Personality, Resources, and Optimism) After the First period of Practice Education Training

With respect to the female students, disregarding the internship area, the results show that the “tough personality” dimension improved significantly after the practical training (2.14 points; *p* < 0.001) (Table 5). Taking the internship area into account, there was an improvement in the score of this dimension in those participants who carried out their practical training in “other areas,” initially a score of (22) is achieved, a score after clinical practice of (25.57), with a difference in means of (3.57 points; *p* = 0.014), in mental health we found a pre-practice score of (24.57), reaching a post-practice score of (27.36) and a mean difference of (2.79 points; *p* = 0.003), in physical rehabilitation we start from an initial score of (24.98), arriving at a final score after clinical practice of (27.46), and a difference in means of (2.48 points; *p* < 0.001), and finally, with regard to the field of geriatrics and gerontology, we start from a previous score of (25.94), reaching a score after the clinical practice of (27.31), achieving a difference in the means of (1.37 points; *p* = 0.019) (Table 5). With regard to the “resources” dimension, there were no significant changes in it, neither in general terms nor by internship area (Table 5). With respect to the “optimism” dimension, disregarding the internship area, the results show that it improved significantly after the practice training (0.99 points; *p* < 0.001) (Table 5). Taking the practice placement education area into account, a significant improvement was found in the score of this dimension in those students who carried out their practical training in physical rehabilitation we found an initial score of (18.29), a score after clinical practice of (19.63), resulting in a mean score difference of (1.34 points; *p* = 0.002) (Table 5).

**Table 5:** Practical training PRE-POST scores of dimensions “Tough Personality,” “Resources,” and “Optimism” (CD-RISC 25) in female students (*n* = 131).

	**“Tough Personality”** **PRE** **Mean (SD)**	**“Tough Personality”** **POST** **Mean (SD)**	**Difference between** **means (SD)**	***p*-value** **(paired *t*-test)**	**95% CI**
**General** (*n =* 131)	25.08 (4.35)	27.22 (4.76)	2.14 (4.06)	** <0.001**	1.43–2.84
**Practice Education Areas**					
Children (*n =* 6)	24.83 (4.12)	26.17 (5.12)	1.34 (1.43)	0.394	−2.34–5.01
Physical rehabilitation (*n =* 41)	24.98 (4.18)	27.46 (5.63)	2.48 (4.14)	** <0.001**	1.18–3.80
Mental health (*n =* 28)	24.57 (4.60)	27.36 (4.17)	2.79 (4.44)	**0.003**	1.06 – 4.51
Geriatrics and gerontology (*n =* 49)	25.94 (4.37)	27.31 (4.55)	1.37 (3.95)	**0.019**	0.23 −2.5
Other areas (*n =* 7)	22 (3.70)	25.57 (2.99)	3.57 (2.76)	**0.014**	1.02–6.12
	“Resources” PRE Mean (SD)	“Resources” POST Mean (SD)	Difference between means (SD)	*p*-value (paired *t*-test)	95% CI
**General** (*n =* 131)	22.25 (3.82)	22.74 (3.50)	0.49 (3.23)	0.086	−0.07–1.05
**Practice Education Areas**					
Children (*n =* 6)	23.17 (3.87)	24.33 (2.88)	1.16 (1.62)	0.504	−3–5.33
Physical rehabilitation (*n =* 41)	22.05 (4.09)	22.59 (4.03)	0.54 (3.20)	0.289	−0.47–1.55
Mental health (*n =* 28)	22.50 (4.19)	23.21 (3.13)	0.71 (3.57)	0.299	−0.67–2.10
Geriatrics and gerontology (*n =* 49)	22.04 (3.45)	22.41 (4.37)	0.37 (3.13)	0.416	−0.53–1.27
Other areas (*n =* 7)	23.14 (3.85)	22.71 (2.69)	−0.43 (2.63)	0.682	−2.87–2.01
	“Optimism” PRE Mean (SD)	“Optimism” POST Mean (SD)	Difference between means (SD)	*p*-value (paired *t*-test)	95% CI
**General** (*n =* 131)	18.73 (3.17)	19.72 (2.75)	0.99 (3.15)	** <0.001**	0.45–1.54
**Practice Education Areas**					
Children (*n =* 6)	18.33 (5.99)	19.50 (3.67)	1.17 (2.21)	0.621	−4.52–6.85
Physical rehabilitation (*n =* 41)	18.29 (2.39)	19.63 (3.10)	1.34 (2.53)	**0.002**	0.54–2.14
Mental health (*n =* 28)	18.93 (2.97)	20 (2.40)	1.07 (3.33)	0.100	−0.22–2.36
Geriatrics and gerontology (*n =* 49)	19.41 (2.89)	19.69 (2.69)	0.28 (2.78)	0.475	−0.51–1.08
Other areas (*n =* 7)	16 (5.42)	19.43 (2.15)	3.43 (4.96)	0.117	−1.16–8.02

*SD, standard deviation; 95% CI, 95% Confidence Interval*.

*The values marked in bold are the result of the analyses shown in the article*.

*These references are significant at a significance of p < 0.05*.

Regarding the male students, there were no significant changes in any of the analyzed dimensions, neither in general terms nor by internship area (Table 6).

**Table 6:** Practical training PRE-POST scores of dimensions “Tough Personality,” “Resources,” and “Optimism” (CD-RISC 25) in male students (*n* = 25).

	**“Tough Personality”** **PRE** **Mean (SD)**	**“Tough Personality”** **POST** **Mean (SD)**	**Difference between means (SD)**	***p*-value** **(paired *t*-test)**	**95% CI**
**General** (*n =* 25)	26.24 (5.04)	26.44 (5.02)	0.20 (4.72)	0.834	−1.75–2.15
**Practice Education Areas**					
Children (*n =* 2)	24.50 (10.61)	18 (2.83)	−6.50 (7.78)	0.447	−76.38–63.38
Physical rehabilitation (*n =* 5)	27.40 (7.34)	27.60 (4.28)	0.20 (3.19)	0.895	−3.77–4.17
Mental health (*n =* 3)	28.33 (2.08)	29 (3.61)	0.67 (1.53)	0.529	−3.13–4.46
Geriatrics and gerontology (*n =* 14)	25.79 (4.30)	26.57 (5)	0.78 (4.95)	0.563	−2.07–3.64
Other areas (*n =* 1)^*^	NA	NA	NA	NA	NA
	“Resources” PRE Mean (SD)	“Resources” POST Mean (SD)	Difference between means (SD)	*p*-value (paired *t*-test)	95% CI
**General** (*n =* 25)	22.76 (3.75)	22.36 (3.32)	−0.40 (3.55)	0.578	−1.86–1.06
**Practice Education Areas**					
Children (*n =* 2)	21.50 (4.95)	17.50 (0.71)	−4 (4.24)	0.410	−42.12–34.12
Physical rehabilitation (*n =* 5)	24.60 (4.51)	24.60 (3.36)	0 (3.81)	1	−4.73–4.73
Mental health (*n =* 3)	24.33 (2.31)	23 (4.36)	−1.33 (4.04)	0.625	−11.37–8.71
Geriatrics and gerontology (*n =* 14)	22 (3.76)	21.93 (2.73)	−0.07 (3.41)	0.939	−2.04–1.90
Other areas (*n =* 1)^*^	NA	NA	NA	NA	NA
	“Optimism” PRE Mean (SD)	“Optimism” POST Mean (SD)	Difference between means (SD)	*p*-value (paired *t*-test)	95% CI
**General** (*n =* 25)	18.56 (3.45)	20.12 (2.79)	1.56 (4.16)	0.073	−0.16–3.28
**Practice Education Areas**					
Children (*n =* 2)	18 (8.49)	14.50 (2.12)	−3.50 (6.36)	0.579	−60.68–53.68
Physical rehabilitation (*n =* 5)	18.60 (2.70)	19.60 (2.07)	1 (1.41)	0.189	−0.76–2.76
Mental health (*n =* 3)	19.67 (3.06)	21 (1.73)	1.33 (2.08)	0.383	−3.84–6.50
Geriatrics and gerontology (*n =* 14)	18.50 (3.50)	21.07 (2.40)	2.57 (4.74)	0.063	−0.16–5.31
Other areas (*n =* 1)^*^	NA	NA	NA	NA	NA

*SD, standard deviation; 95% CI, 95% Confidence Interval*;

* *The correlation and T cannot be calculated because the sum of the weights of the cases is ≤1; NA, Not applicable*.

## Discussion

The aim of this study was to determine whether the first exposure to clinical practice placement education improves the resilience skills of students of occupational therapy. The results of this study show that there are statistically significant differences between the variables of resilience and the different internship areas. According to Ashby et al. (2013), this could be due to some factors that favor resilience, such as: a professional practice based on the use of theory and concepts; different ways of explaining the evidence-based practice depending on the target professionals; the consideration of other theories from other fields, such as psychology; professional socialization, that is, being in contact with other occupational therapists who work in the same professional area; and, lastly, being supervised by another occupational therapist (Ashby et al., 2013). Occupational therapists may foster student veterans' resilience by promoting meaningful activity, social support, and coping ability, developing the motivation.

Likewise, meaningful occupation is a perspective of doing that helps motivate and maintain a constant commitment to daily life through one's occupation, thus offering a protective resource that fosters resilience and personal well-being (Eakman, 2014; Kinney et al., 2020). Therefore, considering these factors, the fact of confronting an external internship for the first time may be a positive and less stressful challenge if the students begin associating the theory with the practice and learn from other professionals and users from the 1st year. This would allow them to develop a professional identity and learn different ways of solving problems in the clinical practice. In this way, when students complete their final years of study, they will be able to be competent to define the theoretical and practical aspects of therapy and develop the necessary coping strategies to manage the stress of working in the profession (Brown et al., 2019).

Another important aspect has been approached by Arghode (2013), who pointed out the need to detect the mood and feelings that emerge when interacting with other people, since this may influence the learning process. This could be applied in the professional context, since there is evidence of how optimistic thoughts and hopeful feelings improve the levels of resilience and personal satisfaction (Glass, 2007; Arghode, 2013). Studies such as that of Williamson et al. (2013) suggest that health professional students who implement effective stress management strategies are more motivated and optimistic about her future, strengthening her professional and social belonging. In our study, we also found significant differences in the optimism dimension, considering the beginning and the end of the practice education. This indicates that a positive experience contributes to the development of confrontation strategies focused on the problem, which is in line with the findings of Kim and Lee (2018). In their study to determine the distinguishing characteristics of the most resilient students, these authors found that this group used specific cognitive and emotional regulation strategies, such as pre-treatment strategic planning with the patient, positive retraining according to each environmental situation, and restructuring the situation with optimistic views. Furthermore, such groups showed greater professional satisfaction compared to the maladaptive group (Kim and Lee, 2018).

These results could suggest that, to a reasonable extent, the exposure to practical training during the university degree could prevent the students of occupational therapy from developing in the future the so-called burnout syndrome in the workplace, of which there is enough scientific evidence from studies carried out with healthcare professionals like nursing and medical (Dunn et al., 2008; Scanlan et al., 2017). As stated by these authors, the well-being of medical students is influenced by multiple stressful factors, as well as positive aspects of medical training; thus, it is fundamental to teach confrontation strategies that promote well-being and minimise fatigue, since the average medical students tend to be very self-demanding, as they aim to be the best of their class during their university studies (Harms et al., 2018; Hayes, 2018).

When this is constant, it can generate a continuous exhaustion, making it necessary for medical schools to help their students to develop resilience, in order to maintain their well-being throughout their studies, since this will be beneficial for medical education, in general, and for their position in the workplace. This would promote resilience at the professional and personal level, which implies an improvement in the quality of the attention to the patient (Dunn et al., 2008), and ultimately in the well-being of the healthcare professionals (Chow et al., 2018).

McDonald et al. (2013) established an educational intervention program for nursery professionals to boost resilience and provide protection against adversity in the workplace. Through participation in an experience-based learning, they practiced creative self-expression and acquired new ideas and strategies, gaining personal improvements (McDonald et al., 2013).

Considering the level of resilience at the first exposure to external practice education, the results obtained suggest that, after the practical training, students improved in resilience-related skills marked in the questionnaire collected on the CD-RISC such as goal setting, behavior, commitment and decision making when unexpected events or situations of uncertainty and frustration occurred. Furthermore, there were also improvements in the global score of resilience when the practice education area was taken into account, with these improvements being statistically significant in the female students within the areas of attention to children, physical rehabilitation, mental health and geriatrics and gerontology. This may be due to a cultural issue. Clauss-Ehlers (2008) found that strong ethnic and gender identities were predictive of resilience so that women develop strategies of resilience from childhood due to the responsibility they have in housework, childcare or elderlycare (Clauss-Ehlers, 2008). As for aspects such as optimism after practical training, a significant improvement indicates a more positive attitude toward adverse or risky situations.

Resilient students are optimistic and work to improve any situation beyond doing simply what is expected of them, knowing how to control their feelings. This is beneficial for students in their practice placement education, since during this practical training they must face difficult situations that require a resilient pattern; understanding the characteristics of the users and knowing each pathology is a challenge that requires certain personality traits. Sheehy et al. (2020) carried out space in the curriculum for five mandatory “Resilience Days” in third year medical students, with corresponding topics to clinical rotations, in which students experienced a positive effect on their mental health and they learned new skills for professional practice.

Therefore, being able to stay calm and overcome a high stress situation can help the practical training, and therefore the situations arising from it, to be carried out in a satisfactory way. Different studies support the relationship between a high level of endurance and greater subjective well-being, which positively influences the quality of life and the perception of the environment of medical science students (Tempski et al., 2015; Bacchi and Licinio, 2017). If the influence of the ability to react to difficult events is analyzed, this implies making decisions about a treatment toward the patient (Houpy et al., 2017).

Houpy et al. (2017) pointed out the importance of delving into resilience in medical students, considering the influence of sociodemographic and cultural aspects. They also mentioned the need to incorporate resilience as a subject matter in the curriculum of the degree of medicine, with the aim of helping students to confront the difficult interactions of the multidisciplinary team. This could also be applied to students of occupational therapy and other disciplines of Health Sciences, since during their training process they go through common stages with interconnected and related duties, confronting vital and critical situations in their healthcare profession (Houpy et al., 2017). It must be taken into account that, in the case of occupational therapists, the interaction with the user is greater, since physicians refer the patients to them and to other professionals, such as physiotherapy, nursing or psychology.

Therefore, since occupational therapists must be in contact with other professionals and interact with users, follow their progress and plan their discharge from occupational therapy services. On the other hand, it is necessary that these professionals acquire a set of personal and professional skills and learn to face unexpected situations and difficulties in a resistant way. In order to do this, they must avoid having their professional practice affect their personal life, and so that they can make the right decision at any time. In this sense, other authors have pointed out the importance of introducing workshops about resilience with the aim of getting students of Health Sciences to know their personal strengths and develop professional skills from the beginning of their university studies (Chow et al., 2018; Gheihman et al., 2018; Lekan et al., 2018). Likewise, through the incorporation of resilience training in the clinical context, educators can better prepare students for the challenges of the educational context and, ultimately, for their practice placement education (Thomas and Asselin, 2018).

This study has some limitations. First, the sample size was small, as it was made up of 2nd and 3rd year students doing an internship for the first time. Second, another limitation was the absence of literature based on previous studies focusing on resistance in occupational therapy students. Thirdly, the inability to determine the strategies used by students to facilitate the development of their resilience. In contrast, there are studies on this topic in nursing and medical students, in which female students predominate in the sample, as in the present study; however, their results are not analyzed by sex and the education of the first practice is not considered.

## Conclusion

The learning of therapeutic skills is a slow and decisive process for the correct development of the professional practice of occupational therapy students. Through the curricular teachings, and more specifically in the external professional practice education, it would be necessary for students to increase their resilience, in order for them to be able to efficiently confront adverse situations in their future workplace. This may also help students to improve their stress management in the face of any new or challenging situation.

The data obtained through this study demonstrates the importance of resilience in health professions, such as occupational therapy, where professionals work with patients affected by different problems and also by the resolution of these. The development of these abilities and skills through the completion of practice placement education poses a significant change in their professional future.

The improvement shown by the students from both the 2nd and 3rd year of the degree of occupational therapy demonstrates an increase of resilience in the first contact with practice placement education, with the pre-post change being very significant, as is shown by the obtained results. Although there were different years and students, the data were treated globally, which showed a significant improvement in both women and men, according to the scores of the global CD-RISC, the “tough personality,” “resources,” and “optimism” dimensions, and their interaction with the practice placement education areas (children, physical rehabilitation, mental health, geriatrics and gerontology, and others). Resilience is an important factor in developing the professional skills of occupational therapists. It also implies a better training in the confrontation of stress, being the practical training fundamental for the development of emotional skills, as indicated in our study.

It is worth highlighting that there are no studies with similar characteristics carried out with students of occupational therapy, both at the national and international level, that analyze the differences regarding gender, exposure to practical training and practice placement education areas.

## Data Availability Statement

The raw data supporting the conclusions of this article will be made available by the authors, without undue reservation.

## Ethics Statement

The studies involving human participants were reviewed and approved by the Ethics Committee of Scientific Research of the Integrated Area of Healthcare in Talavera de la Reina (CEIC code: 6/2017) and by the Ethics Committee of Experimentation of the University of Malaga (CEUMA: 86-2016-H). Likewise, the authors of the original CD-RISC questionnaire authorized the use of this instrument in the present study. The patients/participants provided their written informed consent to participate in this study.

## Author Contributions

All authors listed have made a substantial, direct and intellectual contribution to the work, and approved it for publication.

## Conflict of Interest

The authors declare that the research was conducted in the absence of any commercial or financial relationships that could be construed as a potential conflict of interest.

## References

[B1] Abu-Tineh A. M. (2011). Exploring the relationship between organizational learning and career resilience among faculty members at Qatar University. Int. J. Educ. Manage. 25, 635–650. 10.1108/09513541111159095

[B2] Arghode V. (2013). Emotional and social intelligence competence: implications for instruction. Int. J. Pedag. Learn. 8, 66–77. 10.5172/ijpl.2013.8.2.66

[B3] Ashby S. E. Ryan S. Gray M. James C. (2013). Factors that influence the professional resilience of occupational therapists in mental health practice. Aust. Occupat. Ther. J. 60, 110–119. 10.1111/1440-1630.12012 23551004

[B4] Bacchi S. Licinio J. (2017). Resilience and psychological distress in psychology and medical students. Acad. Psychiatry 41, 185–188. 10.1007/s40596-016-0488-0 27060093

[B5] Bennis W. Cherniss C. Goleman D. (2001). The Emotionally Intelligent Workplace: How to Select For, Measure, and Improve Emotional Intelligence in Individuals, Groups, and Organizations. San Francisco, CA: Jossey-Bass.

[B6] Bonanno G. A. (2004). Loss, trauma, and human resilience: have we underestimated the human capacity to thrive after extremely aversive events? Am. Psychol. 59, 20–28. 10.1037/0003-066X.59.1.20 14736317

[B7] Brown T. Yu M. L. Hewitt A. Isbel S. T. Bevitt T. Etherington J. (2019). Exploring the relationship between resilience and practice education placement success in occupational therapy students. Aust. Occupat. Ther. J. 67, 49–61. 10.1111/1440-1630.12622 31709569

[B8] Chow K. M. Tang W. K. Chan W. H. Sit W. H. Choi K. C. Chan S. (2018). Resilience and well-being of university nursing students in Hong Kong: a cross-sectional study. BMC Med. Educ. 18:13. 10.1186/s12909-018-1119-0 29329529 PMC5767064

[B9] Clauss-Ehlers C. S. (2008). Sociocultural factors, resilience, and coping: support for a culturally sensitive measure of resilience. J. Appl. Dev. Psychol. 29, 197–212. 10.1016/j.appdev.2008.02.004

[B10] Connor K. M. Davidson J. R. (2003). Development of a new resilience scale: the Connor-Davidson Resilience Scale (CD-RISC). Depress. Anxiety 18, 76–82. 10.1002/da.10113 12964174

[B11] Dunn L. B. Iglewicz A. Moutier C. (2008). A conceptual model of medical student well-being: promoting resilience and preventing burnout. Acad. Psychiatry 32, 44–53. 10.1176/appi.ap.32.1.44 18270280

[B12] Eakman A. M. (2014). A prospective longitudinal study testing relationships between meaningful activities, basic psychological needs fulfillment, and meaning in life. OTJR 34, 93–105. 10.3928/15394492-20140211-01 24649934

[B13] Friborg O. Barlaug D. Martinussen M. Rosenvinge J. H. Hjemdal O. (2005). Resilience in relation to personality and intelligence. Int. J. Methods Psychiatr. Res. 14, 29–42. 10.1002/mpr.15 16097398 PMC6878482

[B14] García-León M. A. González-Gómez A. Robles-Ortega H. Padilla J. L. Peralta-Ramírez M. I. (2019). Propiedades psicométricas de la Escala de Resiliencia de Connor y Davidson (CD-RISC) en población española. Anales de Psicología 35, 33–40. 10.6018/analesps.35.1.314111

[B15] García-Vesga M. C. Domínguez-de la Ossa E. (2013). Desarrollo teórico de la resiliencia y su aplicación en situaciones adversas: una revisión analítica. Rev. Latinoamericana de Ciencias Sociales Niñez Juventud 11, 63–77. 10.11600/1692715x.1113300812

[B16] Garner P. W. (2010). Emotional competence and its influences on teaching and learning. Educ. Psychol. Rev. 22, 297–321. 10.1007/s10648-010-9129-4

[B17] Gheihman G. Cooper C. Simpkin A. (2018). Everyday resilience: practical tools to promote resilience among medical students. J. Gen. Int. Med. 34, 498–501. 10.1007/s11606-018-4728-8 30535748 PMC6445902

[B18] Glass N. (2007). Investigating women nurse academics' experiences in universities: the importance of hope, optimism and career resilience for workplace satisfaction. Ann. Rev. Nurs. Educ. 5, 111–136.

[B19] González-Torres M. C. Artuch R. (2014). Resilience and coping strategy profiles at university: contextual and demographic variables. J. Res. Educ. Psychol. 12, 621–648. 10.14204/ejrep.34.14032

[B20] Hanson D. (2015). Closing in the practice–theory gap during fieldwork. OT Practice 20, 13–14.

[B21] Harms P. D. Brady L. Wood D. Silard A. (2018). “Resilience and well-being,” in Handbook of Well-Being, eds Diener E. Oishi S. Tay L. (Salt Lake City, UT: DEF Publishers).

[B22] Hayes C. (2018). Building psychological resilience in the paramedic. J. Paramed. Pract. 10, 147–152. 10.12968/jpar.2018.10.4.147

[B23] Hecht A. D. Fiksel J. Moses M. (2014). Working toward a sustainable future. Sustainability 10, 65–75. 10.1080/15487733.2014.11908133

[B24] Houpy J. C. Lee W. W. Woodruff J. N. Pincavage A. T. (2017). Medical student resilience and stressful clinical events during clinical training. Med. Educ. 22:1320187. 10.1080/10872981.2017.1320187 28460570 PMC5419301

[B25] Ikiugu M. N. Smallfield S. (2015). Instructing occupational therapy students in use of theory to guide practice. Occup. Ther. Health Care 29, 165–177. 10.3109/07380577.2015.1017787 25821888

[B26] Kim S. R. Lee S. M. (2018). Resilient college students in school-to-work transition. Int. J. Stress Manage. 25, 195–207. 10.1037/str0000060

[B27] Kinney A. R. Schmid A. A. Henry K. L. Coatsworth J. D. Eakman A. M (2020). Protective and health-related factors contributing to resilience among student veterans: a classification approach. Am. J. Occupat. Therapy 74, 7404205040–7404205040p11. 10.5014/ajot.2020.038331 32602443

[B28] Lekan D. A. Ward T. D. Elliott A. A. (2018). Resilience in baccalaureate nursing students: an exploration. J. Psychosoc. Nurs. Ment. Health Serv. 56, 46–55. 10.3928/02793695-20180619-06 29975398

[B29] Lutha S. S. Cicchetti D. (2000). The construct of resilience: implications for interventions and social policies. Dev. Psychopathol. 12, 857–885. 10.1017/S0954579400004156 11202047 PMC1903337

[B30] Luthar S. S. Brown P. J. (2007). Maximizing resilience through diverse levels of inquiry: Prevailing paradigms, possibilities, and priorities for the future. Dev. Psychopathol. 19, 931–955. 10.1017/S0954579407000454 17705909 PMC2190297

[B31] Manzano-García G. Ayala Calvo J. C. (2013). Psychometric properties of Connor-Davidson resilience scale in a Spanish sample of entrepreneurs. Psicothema 25, 245–251. 10.1037/t71949-000 23628541

[B32] Martínez-González M. Sánchez-Villegas A. Faulin-Fajardo J. (2009). Bioestad ística amigable, 3rd Edn. Madrid: Díaz de Santos.

[B33] Masten A. S. (2007). Resilience in developing systems: progress and promise as the fourth wave rises. Dev. Psychopathol. 19, 921–930. 10.1017/S0954579407000442 17705908

[B34] McDonald G. Jackson D. Wilkes L. Vickers M. (2013). Personal resilience in nurses and midwives: effects of a work-based educational intervention. Contemp. Nurse 45, 134–143. 10.5172/conu.2013.45.1.134 24099234

[B35] Mestre J. M. Núñez-Lozano J. M. Gómez-Molinero R. Zayas A. Guil R. (2017). Emotion regulation ability and resilience in a sample of adolescents from a suburban area. Front. Psychol. 8:1980. 10.3389/fpsyg.2017.01980 29180978 PMC5693909

[B36] Newman R. (2005). APA's resilience initiative. Prof. Psychol. 36, 227–229. 10.1037/0735-7028.36.3.227

[B37] O'Dougherty W. M. Masten A. N. A. (2013). “Resilience processes in development: four waves of research on positive adaptation in the context of adversity,” in Handbook of Resilience in Children, eds Goldstein S. Brooks R. B. (Boston: Springer US), 15–38. 10.1007/978-1-4614-3661-4_2

[B38] Piña López J. A. (2015). Un análisis crítico del concepto de resiliencia en psicología. Anales De Psicología/Ann. Psychol. 31, 751–758. 10.6018/analesps.31.3.185631

[B39] Rutter M. (2006). Implications of resilience concepts for scientific understanding. Ann. N. Y. Acad. Sci. 1094, 1–12. 10.1196/annals.1376.002 17347337

[B40] Scanlan J. N. Meredith P. J. Haracz K. Ennals P. Pépin G. Webster J. S.. (2017). Mental health education in occupational therapy professional preparation programs: Alignment between clinician priorities and coverage in university curricula. Aust. Occup. Ther. J. 64, 436–447. 10.1111/1440-1630.12397 28660711

[B41] Sheehy J. Yim E. Hayton A. (2020). Third-year resilience days: fortifying students against burnout. Med. Educ. 54, 1051–1052. 10.1111/medu.14341 33006161

[B42] Sinclair V. G. Wallston K. A. (2004). The development and psychometric evaluation of the Brief Resilient Coping Scale. Assessment 11, 94–101. 10.1177/1073191103258144 14994958

[B43] Stoffel V. C. (2014). Attitude, authenticity, and action: building capacity. Am. J. Occup. Ther. 68, 628–635. 10.5014/ajot.2014.686002 25397755

[B44] Tempski P. Santos I. S. Mayer F. B. Enns S. C. Perotta B. Paro H. B.. (2015). Relationship among medical student resilience, educational environment and quality of life. PLoS ONE 10:e0131535. 10.1371/journal.pone.0131535 26121357 PMC4486187

[B45] Thomas L. J. Asselin M. (2018). Promoting resilience among nursing students in clinical education. Nurse Educ. Pract. 28, 231–234. 10.1016/j.nepr.2017.10.001 29128734

[B46] Williamson G. R. Health V. Proctor-Childs T. (2013). Vocation, friendship, and resilience: a study exploring nursing student and staff views on retention and attrition. Open Nurs. J. 7, 149–156. 10.2174/1874434601307010149 24167537 PMC3807580

